# Association mapping for maize stover yield and saccharification efficiency using a multiparent advanced generation intercross (MAGIC) population

**DOI:** 10.1038/s41598-021-83107-1

**Published:** 2021-02-09

**Authors:** A. López-Malvar, A. Butron, R. A. Malvar, S. J. McQueen-Mason, L. Faas, L. D. Gómez, P. Revilla, D. J. Figueroa-Garrido, R. Santiago

**Affiliations:** 1 Facultad de Biología, Departamento de Biología Vegetal y Ciencias del Suelo, Universidad de Vigo, As Lagoas Marcosende, 36310 Vigo, Spain; 2 Agrobiología Ambiental, Calidad de Suelos y Plantas (UVIGO), Unidad Asociada a la MBG (CSIC), Pontevedra, Spain; 3 Misión Biológica de Galicia (CSIC), Pazo de Salcedo, Carballeira 8, 36143 Pontevedra, Spain; 4 Department of Biology, Centre for Novel Agricultural Products, CNAP, University of York, York, YO10 5DD UK

**Keywords:** Biotechnology, Cell biology, Plant sciences

## Abstract

Cellulosic ethanol derived from fast growing C4 grasses could become an alternative to finite fossil fuels. With the potential to generate a major source of lignocellulosic biomass, maize has gained importance as an outstanding model plant for studying the complex cell wall network and also to optimize crop breeding strategies in bioenergy grasses. A genome-wide association study (GWAS) was conducted using a subset of 408 Recombinant Inbred Lines (RILs) from a Multi-Parent Advanced Generation Intercross (MAGIC) Population in order to identify single nucleotide polymorphisms (SNPs) associated with yield and saccharification efficiency of maize stover. We identified 13 SNPs significantly associated with increased stover yield that corresponded to 13 QTL, and 2 SNPs significantly associated with improved saccharification efficiency, that could be clustered into 2 QTL. We have pointed out the most interesting SNPs to be implemented in breeding programs based on results from analyses of averaged and yearly data. Association mapping in this MAGIC population highlight genomic regions directly linked to traits that influence the final use of maize. Markers linked to these QTL could be used in genomic or marker-assisted selection programs to improve biomass quality for ethanol production. This study opens a possible optimisation path for improving the viability of second-generation biofuels.

## Introduction

In a scenario of global growth, depletion of natural resources and climate change, the economic and environmental consequences of reliance in finite fossil biofuels has become a global concern. This situation has driven to exhaustive scientific research in order to find sustainable energetic alternatives. Cellulosic ethanol derived from fast growing C4 crops has become one of the preferred choices due to their high biomass yields, broad geographic adaptation, carbon sequestration and nutrient utilization^1^.

With the potential to generate a major source of lignocellulosic biomass, maize has been postulated as a model for understanding the complex cell wall architecture, and to optimize crop breeding strategies in bioenergy grasses. Maize stover, the residue left after harvesting the grain, is the largest available lignocellulosic feedstock^1,2^.

Lignocellulosic biomass from maize stover is composed of 33.1% hemicellulose, 39.4% cellulose, and 14.9% lignin^3^. The conversion of lignocellulosic biomass to ethanol is a three step process: (i) a pre-treatment stage, followed by the (ii) hydrolytic degradation of carbohydrates to the constituent sugar monomers (saccharification), and the (iii) final fermentation of the free sugars to ethanol^4^.

The key factor in this process is the stover recalcitrance to deconstruction, conferred by the composition and organization of the cell wall. Maize cell walls are mainly composed of cellulose microfibrils embedded in a matrix of hemicelluloses, lignin and to a lesser extent, pectins, proteins and phenolic compounds (mainly hydroxycinnamates)^5^. This strong assemblage provides not only structural support and rigidity to the cell, but also resistance to biotic and abiotic stresses^6^. The framework of hemicellulose and lignin closely interconnected with cellulose prevents the action of hydrolytic enzymes reducing the degradability of carbohydrates. The degree of lignification and the polysaccharides crosslinking by diferulates, as well as cellulose crystallinity contribute to the recalcitrance of lignocellulosic feedstock. This recalcitrance means a greater expense in pre-treatments and high enzyme inputs, which is translated in a greater economic cost. Therefore, reduction of the cell wall recalcitrance is expected to improve saccharification efficiency^7^.

It should be noted that the saccharification properties and subsequent ability to produce ethanol depends on the genotype and on the applied pre-treatment. Therefore, to look for differences for ethanol production among genotypes, it is essential to choose the appropriate treatment for the tissue under study. Among a number of pre-treatments that could be used, alkaline pre-treatment has been suggested as the most appropriate for maize stover and other herbaceous plants^8^. The cell walls of gramineous monocots are known to contain alkali-labile ferulate ester cross-links within the hemicellulose and thereafter cross-linked with lignin^9^, as well as high phenolic hydroxyl contents in their lignin, resulting in increased alkali solubility^10^. As a consequence, mild alkali pre-treatment of grasses can be employed for both fractionating biomass and generating pre-treated biomass that is highly amenable to enzymatic hydrolysis^8^. The optimisation and improvement of stover biofuel production should be focused on stover yields (expressed as tonnes of dry plant material per unit of land area) as well as on the stover quality under a specific pre-treatment.

Mapping QTLs and identifying genes underlying stover quality and quantity are important stages to optimize selection programs for upgrading the biofuel production. Maize genetic variation for saccharification efficiency has been observed^11,12^ and several linkage mapping studies have been conducted to find QTL for saccharification efficiency^13,14^. Furthermore, Trunztler et al.^15^ performed a metaQTL analysis that included several QTL mapping studies for digestibility and cell-wall components and found 27 saccharification-related QTL. Lorenzana et al.^14^ evaluated testcrosses of 223 maize recombinant inbred lines derived from B73 × Mo17 (IBM population) for cell wall composition and glucose release after acid pre-treatment and enzymatic hydrolysis and identified 10 QTL for sugar release, 5 of them co-localizing with QTL for lignin content. Also, in the IBM population per se, Penning et al.^16^ found 4 QTL for saccharification efficiency, measured as glucose or xylose releases after steam explosion, but none of them overlapped with QTL for lignin. The differences in the results found in both studies may be dependent on the heterozygosis level, the pre-treatment chemistry and/or the genotype response to pre-treatment and hydrolysis. Lorenzana et al.^14^ measured the sugar release after dilute acid/high temperature pre-treatment. This method uses strong acids to hydrolyse the hemicellulosic fraction of the biomass, resulting in a more effective enzymatic hydrolysis^17^, whereas in Penning et al.^16^ samples were subjected to steam explosion at 180 °C.

However, the explored genetic variation for saccharification efficiency has been low because the studies mentioned above were performed using just bi-parental populations and thereby the resolution of the detected QTL was low. One of the most robust techniques for high resolution mapping of QTLs is Genome-Wide Association Mapping using diversity panels. This technique has been extensively used in maize to identify significant associations with yield and agronomic traits^18^, biotic and abiotic resistance^6^, cell wall components^19^ and lignin abondance and sugar yield^16^. However, association studies using diversity panels could still have a limited power to detect QTLs with small effects and/or minor alleles in low frequencies (rare alleles). Moreover, many undetected rare alleles could be lost for breeding purposes even if having major effects^20^. Therefore, results from QTL mapping in MAGIC populations could be complementary to results from bi-parental populations and association mapping panels because multiple alleles can be simultaneously studied without any them being in low frequency^21–23^.

We developed a MAGIC population using eight temperate maize inbred lines of diverse genetic origin, the eight founders have a common characteristic: the lack of Stiff Stalk materials in their pedigrees. Therefore, the new inbred lines developed from this MAGIC population could have practical interest for breeders as they are expected to express high heterosis when crossed with inbreds from the Stiff Stalk heterotic group^21,23^. Six founders were directly obtained from different open-pollinated varieties from Spain, Italy, and France, while two inbred lines derived from North American varieties. In the present study, we identified genomic regions and genes putatively associated with saccharification efficiency and stover yield using this MAGIC population. Results provide a better understanding of the genetic factors that can modulate these traits and the molecular tools to be used in breeding programs for increasing stover production and saccharification efficiency.

## Results

A subset of 408 RILs of the MAGIC population together with the eight founders were evaluated for saccharification efficiency of maize stover after alkaline pre-treatment and for stover yield.

### Means and analysis of variance

**Figure 1:**
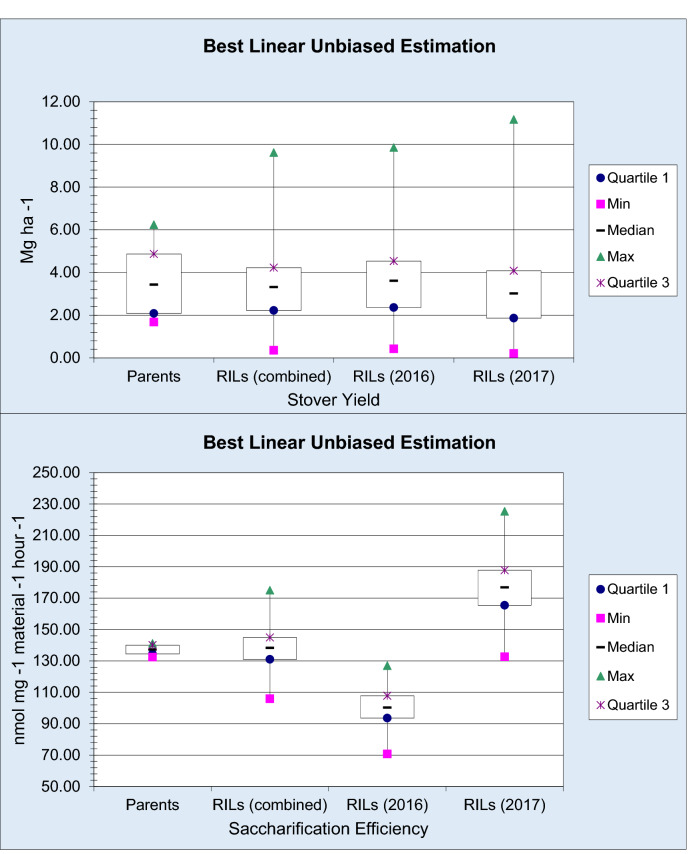
Saccharification efficiency and stover yield values in RILs (combined and by years) and parent lines of the MAGIC population (Best Linear Unbiased Estimation).

The analyses of variance showed that differences among founder inbreds were significant for stover yield but not for saccharification efficiency. However, RIL means differed significantly for both traits without showing year x environment interaction. In addition, data on the main agronomic traits showed that founders and RILs differed significantly for plant height and for days to silking and anthesis. Means and ranges for the traits under study are detailed in Table 1 and in Fig. 1. The phenotypic database is provided as supplementary material (Supplementary Table S1). Pearson correlations between agronomic traits and stover harvesting year showed values lower than 0.50, and thus were not considered relevant (data not shown).

**Table 1:** Average and range values for saccharification efficiency, stover yield and agronomic traits in RILs and parent lines of the MAGIC population.

	Saccharification (nmol/mg material hour)	Stover yield (Mg/ha)	Plant height (2016) (cm)	Days to anthesis	Days to silking
**RILs**					
Means	138.4 ± 1.8	3.3 ± 0.3	131.7 ± 4.5	82 ± 1	86 ± 1
Range	105.9–175.1	0.4–9.6	46.6–210.3	72–96	71–108
LSD (P < 0.05)	22.3	2.67	51.59	11	9
**Founder inbreds**					
A509	139.4 ± 37.9^a^	2.09 ± 0.6^a^	127.8 ± 6.2^a^	82 ± 2^a^	84 ± 2^a^
EP125	137.8 ± 38.0^a^	1.68 ± 0.6^a^	150.0 ± 6.9^ab^	77 ± 2 ^ab^	79 ± 2^ab^
EP17	132.8 ± 37.9^a^	5.82 ± 0.6^b^	124.8 ± 6.5^b^	95 ± 3^b^	101 ± 2^bc^
EP53	135.7 ± 38.1^a^	2.04 ± 0.7^a^	124.4 ± 6.2^b^	82 ± 2 ^b^	83 ± 3^bc^
EP86	141.4 ± 37.9^a^	2.78 ± 0.7^a^	129.2 ± 6.5^b^	85 ± 2^b^	87 ± 4^bc^
F473	140.6 ± 38.3^a^	2.98 ± 0.9^a^	126.6 ± 6.2^b^	84 ± 2 ^b^	89 ± 3^bc^
PB130			136.6 ± 6.2^b^	87 ± 4 ^b^	90 ± 5^c^

^1^Values followed by the same letter within the same column are not significantly different (P > 0.05).

### Association analysis

**Figure 2:**
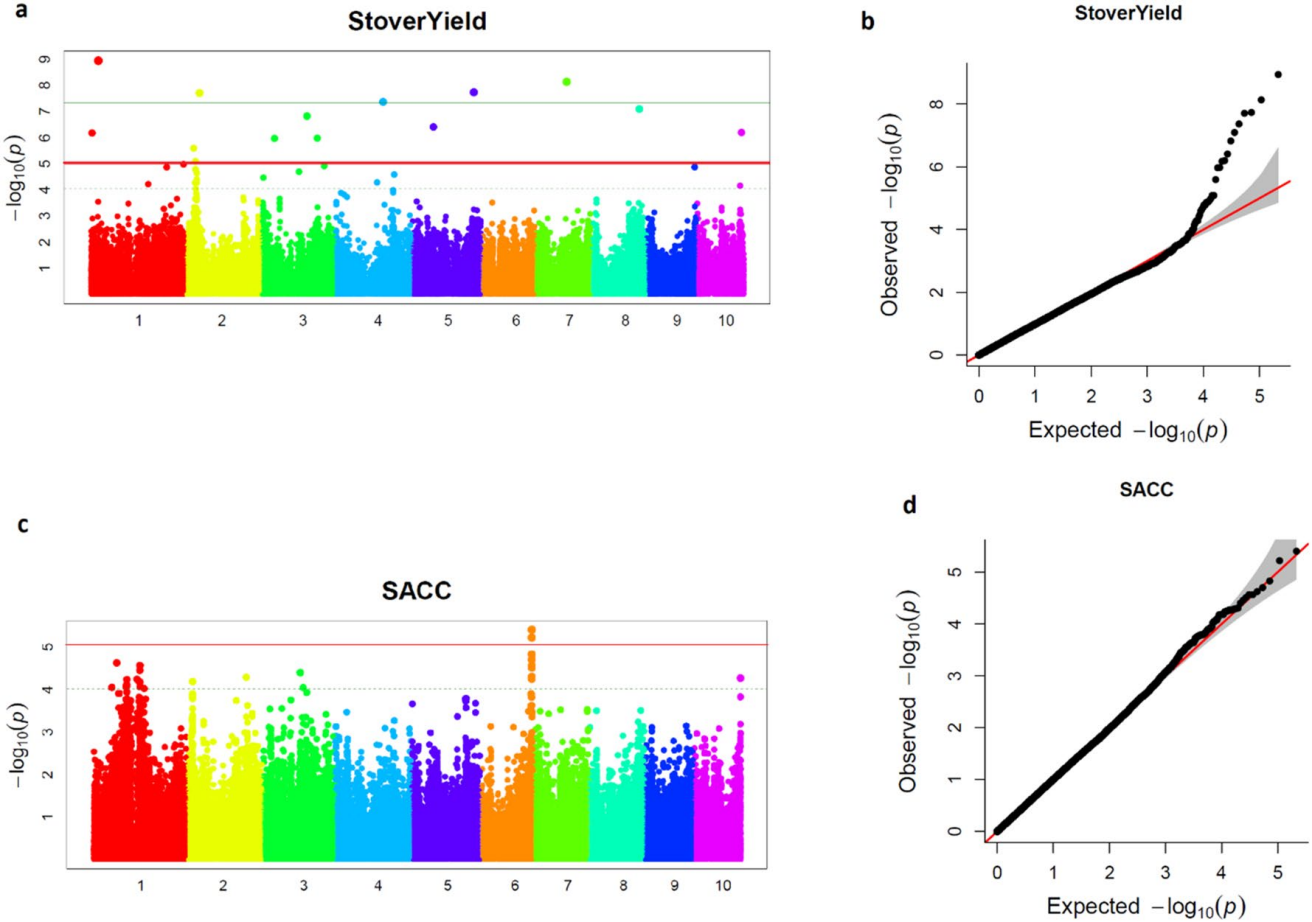
GWAS of stover yield and saccharification efficiency in a maize MAGIC population. (**a**) Manhattan plot of the GWAS mixed linear model for Stover Yield. Single-nucleotide polymorphisms (SNPs) above the red horizontal line surpassed the p value threshold obtained by Bonferroni’s modification approach. (**b**) Quantile–quantile (Q–Q) plot of the GWAS mixed linear model for stover yield. (**c**) Manhattan plot of the GWAS mixed linear model for saccharification efficiency (SACC). Single-nucleotide polymorphisms (SNPs) above the red horizontal line surpassed the p value threshold obtained by the modification of Bonferroni approach (8.07E−06); (**d**) Q-Q plot of the GWAS mixed linear model for SACC.

We carried out GWAS to determine genomic regions that modify stover yield and saccharification efficiency. Q-Q plots showed a good fit of the GWAS models used for saccharification efficiency of the stover and for stover yield (Fig. 2b,d). Reliable associations between some SNPs and stover yield are expected since, in the Q-Q plot, lower *p* values significantly differ from those expected under no significant association. However, associations between SNPs and saccharification efficiency are weaker because none of the SNPs were located in the upper part of the Q-Q plot outside of the 95% confidence interval for no significant association. However, we found two SNPs that presented *p*-values below the threshold (8.07 × 10E−6) established following the modification of Bonferroni’s method (Fig. 2c). For stover yield, 13 SNPs exceeded the *p*-value threshold obtained by applying the modification of the Bonferroni approach (Fig. 2a, Table 2). As expected under no RIL × year interaction, favourable alleles for QTL detected across years also had positive additive effects on the corresponding trait in each year (Table 2). Fifteen SNPs were significantly associated with the traits under study in the two-year combined analysis, 13 of them being also significant in 2016 and nine in 2017 (Table 2). In general, minor alleles were favourable for increasing stover yield except at SNPs S6_163830244, S3_40940154, S7_100264894, and S10_141557921. Favourable alleles for SNPs significantly associated to saccharification efficiency had allele frequencies close to 0.5.

**Table 2:** SNPs significantly associated with stover saccharification efficiency (SACC) and stover yield, including the QTL interval, p-value for the association between the SNP and the phenotype, additive effect for the SNP (across years, 2016, 2017), parental lines alleles, minor and major frequency alleles and favourable allele.

Trait ^a^	SNP^b^	Bin^c^	QTL interval	P-value^d^	R^2^	Add effect ^e^	Add effect 2016 f.	Add effect 2017^ g^	A509	EP125	EP17	EP43	EP53	EP86	F473	PB130	Minor	Major	Effect ( +)
Stover Yield	S1_24739947	1.02	± 500 kbp	1.14E−09	0.001	0.16**	0.03	0.31*	C	T	N	C	C	C	C	C	40	180	Minor (T)
Stover Yield	S1_5087216	1.01	± 100 kbp	6.64E−07	0.04	0.46***	0.46**	0.41**	G	G	G	G	G	G	C	G	29	224	Minor (C)
Stover Yield	S2_23558946	2.03	± 700 kbp	2.54E−06	0.03	0.46**	0.36	0.64**	G	G	N	N	G	N	N	G	13	198	Minor (T)
Stover Yield	S2_42097780	2.04	± 1.2 Mbp	1.96E−08	0.02	0.34**	0.31*	0.10	T	C	T	C	C	C	C	N	15	204	Minor (T)
Stover Yield	S3_40940154	304	± 2Mbp	1.07E−06	0.03	0.49**	0.47*	0.39	A	G	N	A	A	A	A	A	20	184	Major (A)
Stover Yield	S3_143233104	3.05	± 1.5 Mbp	1.50E−07	0.07	0.53***	0.47***	0.48***	C	N	N	C	C	G	C	G	62	248	Minor (G)
Stover Yield	S3_175552327	3.06	± 300 kbp	1.05E−06	0.04	0.46**	0.48*	0.29	G	A	G	N	G	A	A	G	14	162	Minor (A)
Stover Yield	S4_150637846	4.05	± 600 kbp	4.29E−08	0.04	0.56**	0.61**	0.29	G	G	N	N	G	N	N	G	16	177	Minor (A)
Stover Yield	S5_67669376	5.03	± 700 kbp	3.90E−07	0.04	0.38**	0.49***	0.23	C	C	A	C	C	N	C	C	47	168	Minor (A)
Stover Yield	S5_194837018	5.05	± 700 kbp	1.84E−08	0.05	0.37***	0.35**	0.33**	T	T	T	G	G	G	G	T	85	129	Minor (T)
Stover Yield	S7_100264894	7.02	± 800 kbp	7.32E−09	0.02	0.25**	0.28**	0.20	G	G	C	C	C	C	C	G	105	153	Major (C)
Stover Yield	S8_152321302	8.06	± 900 kbp	8.00E−08	0.06	0.40***	0.41***	0.33**	C	C	C	A	A	A	A	C	72	167	Minor (A)
Stover Yield	S10_141557921	10.06	± 100 Kbp	6.32E−07	0.07	0.42***	0.25*	0.41***	N	G	N	G	G	G	N	G	78	99	Major (G)
SACC	S6_163628712	6.07	± 100 Kbp	6.04E−06	0.09	3.11***	1.50*	4.14***	A	T	N	N	A	T	N	T	102	105	Minor (T)
SACC	S6_163830244	6.07	± 100 Kbp	3.97E − 06	0.08	2.90***	2.22***	2.90*	C	C	C	C	N	C	C	T	116	119	Major (T)

*p < 0.05.

**p < 0.01.

***p < 0.001.

^a^*SACC* Saccharification efficiency.

^b^The number before the underscore indicates the chromosome number and the number after the underscore indicates the physical position in bp within the chromosome.

^c^A bin is the interval that includes all loci from the leftmost or top Core Marker to the next Core Marker. The genetic maps are divided into 100 segments of approximately 20 centiMorgans designated with the chromosome number followed by a two-digit decimal.

^d^p-value obtained in FarmCPU.

^e,f,g^The Additive ffect Add effect)—combined (e), 2016 (f) and 2017 (g), ): was calculated in TASSEL as half the difference between the mean of the homozygous for the allele with the largest value and the mean of the homozygous for the allele with the smallest value.

All founders carry at least one favourable allele for stover yield QTL. Therefore, pyramiding all favourable alleles seems a promising procedure to deliver inbreds with enhanced characteristics compared to each individual founder. In addition, most inbreds, except EP43 and EP53, seem to bring useful rare alleles for increasing stover yield. On the other hand, half of the founders brought useful alleles for improving saccharification efficiency and no rare useful variants for this trait have been found. The approximate confidence interval (± distance from the SNP) for each significant SNP was established as the minimum distance between SNPs at which the non-linear regression line based on the rift-recombination model (Hill & Weir, 1988) predicts an r^2^ estimate below 0.2. The genes containing or physically close (within the SNP confidence interval (CI) when CI ≤ 1 Mbp) to SNPs significantly associated with traits were identified and characterized according to the maize B73 reference genome assembly, version 4 (Supplementary Table S2). No candidate genes for stover yield and saccharification efficiency could be highlighted.

## Discussion

An optimisation of maize stover as a biofuel feedstock can be achieved using plant breeding to increase stover yield and quality. Stover quality is associated to the release of fermentable sugars which is greatly affected by cell wall composition^24^. The deployment of molecular tools to comprehend genomics involved in such complex traits as well as the possible use of these tools in breeding programs to speed up selection is heavily recommended. Multiparental populations constituted by founders with useful complementary alleles could be excellent sources of base breeding materials as well as optimal populations for QTL mapping. The increased levels of genetic diversity and lower linkage disequilibrium compared to bi-parental populations, and higher frequencies of minor alleles than in inbred panels give MAGIC populations a good balance between QTL mapping power and resolution. In addition, even though QTL resolution in MAGIC populations is not as high as in diversity panels, MAGIC populations present a known underlying structure that prevents from false positive associations than unstructured populations^25^. Therefore, results from QTL mapping in MAGIC populations could be complementary to results from bi-parental populations and association mapping panels. Besides, in general, MAGIC populations are adapted to a particular environment and phenotyping at that environment is not adversely affected by lack of acclimatization of some individuals. On the contrary, adaptation differences among inbreds of association panels may impair the phenotyping.

The MAGIC population used in this study seems a promising alternative for QTL mapping and for selecting no Stiff Stalk Synthetic inbreds with increased general combined ability for stover saccharification efficiency and yield because wide genetic variation has been found for both traits among the population RILs. RILs showing the best values for each trait could be either tested for combining ability with specific testers in order to generate promising hybrids or used as base material of recurrent selection programs. One of the main characteristics of this population is the lack of Stiff Stalk germplasm; thereby, we could expect good combining ability between these RILs and inbreds from the Stiff Stalk heterotic group. Therefore, GWAS in this MAGIC population could give an insight for better understanding of the genetic factors that can modulate stover yield and saccharification efficiency and the genetic relationship between them in order to obtain varieties with the best profitability for maize stover. More interestingly, it could render molecular tools and useful and adapted materials for selection program implementation. The significant SNPs associated both with saccharification efficiency and stover yield could be applied in breeding programs for increasing stover biofuel production in areas with climatic characteristics similar to those of northwest Spain where maize can be cultivated without irrigation. In both years, minimum, maximum and average temperatures were around 8, 20 and 14 °C but years differed for mean accumulated precipitation (1486 and 966 mm in 2016 and 2017, respectively)^26^.

None of the genetic markers significantly associated with saccharification efficiency in this study coincides in the same genetic “bin” with those previously described for glucose or saccharification yield measured as sugar release. None of the markers significantly associated with saccharification efficiency, located in bin 6.07 in this study, coincides in the same bin with those previously described for saccharification yield in the IBM population^14^. Barriére et al.^27^, in a RIL progeny derived from the cross between a WM13 and RIo, found three QTL associated with stover (plant without ear), one of them in the same bin position as S3_143233104, significantly associated with stover yield in our study. On the other hand, we could not find any other association study which explores genomic regions associated with stover yield. Therefore, our study represents novel contribution for a double exploitation, since maize ethanol production from the lignocellulosic material does not compete with kernel production for feed and food purposes.

We would recommend a breeding strategy based on genomic selection for increasing simultaneously stover yield and saccharification efficiency. Genomic selection is preferred over marker-assisted selection because the variation for saccharification efficiency was poorly explained by the significant SNPs found. Another possible strategy is phenotypic selection combined with marker assisted selection for rare alleles at stable SNPs across environments for stover yield and significant SNPs for saccharification efficiency. Stable SNPs would be those with similar and significant (p < 0.05) additive effects in both years.

Regarding candidate genes, within the confidence intervals stablished following LD decay^28–30^ we don’t find any suitable genes for the traits under study.

## Conclusion

Association mapping in this MAGIC population allows us to highlight genomic regions directly linked to traits that influence final use of maize. Genomic or phenotypic selection combined with marker-assisted selection using markers located in those regions would help to develop plant materials for higher biofuel yield. This study opens a possible optimisation path for improving the viability of second-generation biofuels.

## Material and methods

### Plant material

The founders of the MAGIC population were the inbred lines EP17, EP43, EP53, EP86, PB130, F473, A509, and EP125. Procedures used to release the 672 recombinant inbred lines of the population have been previously reported^23,31^.

### Experimental design and statistical analysis

A subset of 408 RILs of the MAGIC population together with the eight founders were tested in a single augmented design with 10 blocks in Pontevedra, Spain (42° 24′ N, 8° 38′ W and 20 m above sea level), in two years (2016 and 2017). In each block, 42 non replicated RILs were included, along with the eight inbred founders of the MAGIC population. The founder PB130 was replaced by EC212 in 2017 and the founder EP43 was replaced by EP80 in both years due to lack of seed availability. Only 30 RILs were evaluated in block 10. Each experimental plot consisted of a single row with 13 single-kernel hills planted manually, spacing between consecutive hills in a row being 0.18 m and 0.8 m between rows, obtaining a final density of ~ 70,000 plants ha^−1^. Local agronomical practices were followed.

Data from both trials (2016 and 2017) were combined according to the mixed model procedure (PROC MIXED) of the SAS program (version 9.4)^34^ and the best linear unbiased estimator (BLUE) for each inbred line was calculated based on the combined data for the 2-year analysis. Lines were considered as fixed effects, while years and blocks within years were treated as random effects. BLUEs per year for each inbred line were also estimated. The comparison of means was carried out using the Fisher’s protected least significant difference (LSD) test. Pearson correlations were performed between stover yield and the agronomic traits.

#### Agronomic traits

Days to silking/anthesis was considered as the time from the day of sowing until approximately 50% of the plants showed either pollen (male anthesis) or silks (female silking). Plant height represents the mean of plant height of five plants per plot, measured from the base of the plant to the flag leaf.

#### Stover yield and saccharification determinations

Plots were harvested approximately 55 days after silking (days from planting until half of the plants in the plot showed visible silks). In each plot, the weight of plants without ears (weight of fresh stover) was recorded, and a stover sample was collected for estimating the percentage of stover dry matter and carry out the saccharification efficiency analyses. The stover sample was composed of tissue from two to ten plants, the fresh stover was weighed (sample fresh weight), chopped, pre-dried at 35 °C in a fan-assisted chamber, dried at 60 °C in a stove and again weighed (sample dry weight). Dry stove samples from each plot were ground in a Wiley mill with a 0.75 mm screen for saccharification assays.

Stover yield in Mg ha^−1^ was determined by the following equation:

$$Stover \,\, yield=\frac{weigth\,\,of\,\,fresh\,\,stover \,\,\left(g\right)\,\,*\,\,sample \,\, dry\,\, weight\,\,\left(g\right)*100}{0.144m2*number\,\, of\,\, harvested\,\, plants*sample\,\, fresh\,\, weight\,\,\left(g\right)}$$

where weight of fresh stover is the weight of two to ten plants without ear of each plot measured at harvest; sample fresh weight is the weight of a representative sample of tissue from two to ten plants without ear chopped from each plot measured at harvest; sample dry weight is the weight of sample fresh weight after the sample is pre-dried at 35 °C in a fan-assisted chamber and then dried at 60 °C in a stove; 0.144 corresponds to the surface calculated as the number of harvested plants per plot multiplied by the space between rows (0.80 m) and the space between plants (0.18 m). Yields were calculated per plant and then transformed to Mg/ha; thus, it refers to a maximum yield and not to the yield in a plot.

Saccharification assays were performed as described in Gomez et al.^32^. Ground material was weighed into 96-well plates, each well contained 4 mg of each sample (four replicates/sample), and processed using a high-throughput automated system (Tecan). Samples were pre‐treated with 0.5 M NaOH at 90 °C for 30 min, washed four times with 500 μl sodium acetate buffer and finally subjected to enzymatic digestion (Celluclast 2, 7FPU/g) at 50 °C for 9 h. The amount of released sugars was assessed against a glucose standard curve using the 3-methyl-2-benzothiazolinone hydrozone method^33^.

### Association mapping

Inbred lines were previously genotyped using a genotyping-by-sequencing (GBS) strategy for 955,690 SNPs at the Institute of Biotechnology of the Cornell University. Genotypic and phenotypic datasets were combined. SNPs with more than 50% missing data and/or minor allele frequency less than 5% were omitted, as well as monomeric and multi-allelic SNPs and insertion/deletion polymorphisms (INDELs). Heterozygous genotypes were considered missing data. After filtering, 215,131 SNPs distributed across the maize genome were retained. Complete and filtered genotype databases are available as supplementary materials.

A genome-wide association study (GWAS) of the BLUEs across years was carried out with FarmCPU^35^ based on a mixed linear model using R. The model included the genotypic data (GD), phenotypic data (Y) and marker physical map (GM). The MLM model included the VanRaden’s Kinship matrix and no covariates. Following the developers’ recommendations, we increased the number of iterations (maxLoop = 10) in FarmCPU to boost the power/false discovery rate and we optimized bin size and bin selection (default set, method.bin = "optimum") that are related to the linkage disequilibrium (LD) distance. Manhattan and Q-Q plots were generated with this function. The script’s details are included as supplementary material^35^.

The *p* value threshold to label an association as significant was calculated by a modification of the Bonferroni’s approach using the effective number of independent markers (= number of linkage blocks) as correction factor instead of the total number of markers and assuming and experiment wise error of 0.10. Linkage blocks were determined using the Haploview software^36^ with the solid spine method of linkage disequilibrium (“solid spine of LD”) with D’ > 0.20. This method developed by Barrett^36^ defines LD blocks in which the first and last markers in the block are in strong LD with all intermediate markers but in which the intermediate markers are not necessarily in LD with each other. Haploview produced 12,397 independent blocks. Then, the assumed experiment-wise threshold (0.10) was divided by the number of independent linkage blocks resulting in a comparison-wise *p* value threshold of 8.07E-6^37,38^. The Additive effects in each year and across years of the SNPs significantly associated with a trait was calculated in TASSEL^39^ as half the difference between the mean of the homozygous for the allele with the largest value and the mean of the homozygous for the allele with the smallest value.

LD was estimated by calculating the square value of correlation coefficient (r^2^) between all pairs of markers in TASSEL. In a window of ± 5Mbp around each significant SNP, the LD (r^2^) decay with distance was fitted using a non-linear regression model. The drift-recombination model^30^ was used to fit a nonlinear regression of the expectation of r^2^, using the R script from Marroni et al.^28^ based on the equation described by Remington et al.^29^.

The approximate confidence interval for each SNP that presented p values below the threshold obtained by the modification of Bonferroni´s approach was established by checking the LD decay limits. To visualize LD decay, the r^2^ data on marker-pair distance in a 10 Mbp window around each significant marker was plotted (Supplementary Fig. S1); limits of such confidence interval being the left and right physical positions from where LD estimates (r^2^) drop below 0.2. SNPs with overlapping confidence intervals were clustered in the same QTL. The approximate confidence interval (± distance from the SNP) for each significant SNP was established as the minimum SNP-pair distance at which the nonlinear regression line based on the drift-recombination model^30^ predicts an r^2^ estimate below 0.2. The search for candidate genes was performed within SNP confidence intervals. The SNP was positioned in version 4 (v4) of the maize genome to establish the confidence interval. All genes contained in the confidence intervals were then identified and characterized based on the maize B73 reference genome assembly (v4) available on the MaizeGDB browser. All genes are detailed in Supplementary Table S2.

## Supplementary Information

Supplementary Information 1.

Supplementary Information 2.

Supplementary Information 3.

Supplementary Information 4.

Supplementary Information 5.

Supplementary Information 6.

Supplementary Information 7.

## Data Availability

The data sets used and/or analysed during the current study are available as supplementary material. Vegetal materials are distributed to the scientific community by Maize Genetics and Breeding group of MBG-CSIC upon request (http://www.mbg.csic.es/en/plant-genetics-and-breeding-department/maize-genetics-and-breeding/. RA Malvar, rmalvar@mbg.csic.es).

## Abbreviations

MAGIC: Multi-Parent Advanced Generetion InterCross
GWAS: Genome wide association study
SNP: Single nucleotide polymorphism
QTL: Quantitative Trait Loci
LD: Linkage disequilibrium
SACC: Saccharification efficiency
RIL: Recombinant inbred line
CI: Confidence interval
