# Supplementary material for: Association mapping for maize stover yield and saccharification efficiency using a multiparent advanced generation intercross (MAGIC) population
Source: Sci Rep. 2021 Feb 9;11:3425. doi: 10.1038/s41598-021-83107-1 (PMC7873224; doi:10.1038/s41598-021-83107-1)
Supplement: Supplementary file 5 — Supplementary Information 5. [file 41598_2021_83107_MOESM5_ESM.docx]

**Association Mapping for Maize Stover Yield and Saccharification Efficiency Using a Multi-Parent Advanced Generation Intercross (MAGIC) Population**

López-Malvar, A^1^*; Butron A^3^; Malvar, RA^3^; Gómez, LD^2^; Faas, L^2^; McQueen-Mason, S^2^; Revilla P^3^; Figueroa-Garrido, DJ^1^; Santiago, R^1^

^1^Facultad de Biología, Departamento de Biología Vegetal y Ciencias del Suelo, Universidad de Vigo, As Lagoas Marcosende, Vigo 36310, Spain. Agrobiología Ambiental, Calidad de Suelos y Plantas (UVIGO), Unidad Asociada a la MBG (CSIC);

^2^Misión Biológica de Galicia (CSIC), Pazo de Salcedo, Carballeira 8, 36143, Spain

^3^CNAP, Department of Biology, University of York, Heslington, York YO10 5YW, UK

*Corresponding author: [alopezmalvar@uvigo.es](mailto:alopezmalvar@uvigo.es)

**Supplementary Table 2: List of annotated genes within the QTL interval around each SNP significantly associated with saccharification efficiency and stover yield in a MAGIC population.**

| Trait^a^ | SNP^b^ | Position | Chr^c^ | Bin^d^ | Gene | Gene Function |
| --- | --- | --- | --- | --- | --- | --- |
| Stover Yield | S1_24739947 | 24739947 | 1 | 1.02 | Zm00001d028159 | F-box protein GID2 |
|  |  |  |  |  | Zm00001d028160 | CASP-like protein 2B2 |
|  |  |  |  |  | Zm00001d028161 | hypothetical protein |
|  |  |  |  |  | Zm00001d028162 | sfp1 - sulfate permease1 |
|  |  |  |  |  | Zm00001d028164 | sfp3 - sulfate transporter3 |
|  |  |  |  |  | Zm00001d028165 | dapat3 - diaminopimelate aminotransferase3 |
|  |  |  |  |  | Zm00001d028167 | Cytochrome b561 and DOMON domain-containing protein |
|  |  |  |  |  | Zm00001d028168 | hypothetical protein |
|  |  |  |  |  | Zm00001d028169 | Plant mobile domain protein family |
|  |  |  |  |  | Zm00001d028170 | Dopamine beta-monooxygenase |
|  |  |  |  |  | Zm00001d028171 | Calcium-dependent lipid-binding |
| Stover Yield | S1_5087216 | 5087216 | 1 | 1.01 | Zm00001d028172 | spermidine oxidase |
|  |  |  |  |  | Zm00001d028173 | FRIGIDA-like protein 1 |
|  |  |  |  |  | Zm00001d028174 | hypothetical protein |
|  |  |  |  |  | Zm00001d028175 | Pumilio domain-containing protein |
|  |  |  |  |  | Zm00001d028176 | hypothetical protein |
|  |  |  |  |  | Zm00001d028177 | Ubiquitin carboxyl-terminal hydrolase 26 |
|  |  |  |  |  | Zm00001d028178 | nactf64 - NAC-transcription factor 64 |
|  |  |  |  |  | Zm00001d028179 | hypothetical protein |
|  |  |  |  |  | Zm00001d028180 | mips1 - myo-inositol phosphate synthase1 |
|  |  |  |  |  | Zm00001d027427 | uce3 - ubiquitin conjugating enzyme3 |
|  |  |  |  |  | Zm00001d027428 | Transmembrane 9 superfamily member 11 |
|  |  |  |  |  | Zm00001d027429 | phragmoplastin interacting protein 1 |
|  |  |  |  |  | Zm00001d027430 | Protein FIZZY-RELATED 1 |
|  |  |  |  |  | Zm00001d027431 | knox1 - knotted related homeobox1 |
|  |  |  |  |  | Zm00001d027434 | Alpha/beta hydrolase related protein |
|  |  |  |  |  | Zm00001d027435 | hb30 - Homeobox-transcription factor 30 |
| Stover Yield | S2_23558946 | 23558946 | 2 | 2.03 | Zm00001d002830 | ivr1 - invertase1 |
|  |  |  |  |  | Zm00001d002834 | Peroxisomal biogenesis factor 11 family protein |
|  |  |  |  |  | Zm00001d002835 | putative LRR receptor-like serine/threonine-protein kinase |
|  |  |  |  |  | Zm00001d002836 | downstream target of AGL15 2 |
|  |  |  |  |  | Zm00001d002837 | Transmembrane ascorbate ferrireductase 1 |
|  |  |  |  |  | Zm00001d002839 | Nuclear pore complex protein NUP96 |
|  |  |  |  |  | Zm00001d002841 | hypothetical protein |
|  |  |  |  |  | Zm00001d002842 | remo3 - remorin3 |
|  |  |  |  |  | Zm00001d002843 | myb54 - MYB-transcription factor 54 |
|  |  |  |  |  | Zm00001d002844 | limtf6 - LIM-transcription factor 6 |
|  |  |  |  |  | Zm00001d002845 | (+)-neomenthol dehydrogenase, partial |
|  |  |  |  |  | Zm00001d002846 | late embryogenesis abundant protein |
|  |  |  |  |  | Zm00001d002847 | (+)-neomenthol dehydrogenase |
|  |  |  |  |  | Zm00001d002848 | (+)-neomenthol dehydrogenase |
|  |  |  |  |  | Zm00001d002849 | CLE family OsCLE403 protein |
|  |  |  |  |  | Zm00001d002850 | Elicitor-responsive protein 3 |
|  |  |  |  |  | Zm00001d002851 | UDP-D-galacturonate:1,4-a-poly-D-galacturonate 4-a-D-galacturonosyltransferase |
|  |  |  |  |  | Zm00001d002852 | Sucrose synthase 3 |
|  |  |  |  |  | Zm00001d002853 | putative beta-D-xylosidase 6 |
|  |  |  |  |  | Zm00001d002854 | Thioredoxin M1 chloroplastic |
|  |  |  |  |  | Zm00001d002856 | E3 ubiquitin-protein ligase RMA1 |
|  |  |  |  |  | Zm00001d002857 | OSJNBa0074L08.19-like protein |
|  |  |  |  |  | Zm00001d002859 | D-glucosyl-N-acylsphinganine 4-desaturase |
|  |  |  |  |  | Zm00001d002860 | Expansin-B3 |
|  |  |  |  |  | Zm00001d002862 | casein kinase 1-like protein 2 |
|  |  |  |  |  | Zm00001d002863 | Single-stranded nucleic acid binding R3H protein |
|  |  |  |  |  | Zm00001d002864 | putative polyol transporter 4 |
|  |  |  |  |  | Zm00001d002865 | Transducin/WD40 repeat-like superfamily protein |
|  |  |  |  |  | Zm00001d002866 | hypothetical protein |
|  |  |  |  |  | Zm00001d002867 | ereb154 - AP2-EREBP-transcription factor 154 |
|  |  |  |  |  | Zm00001d002868 | ferredoxin/thioredoxin reductase subunit A (variable subunit) 2 |
|  |  |  |  |  | Zm00001d002869 | uncharacterized |
|  |  |  |  |  | Zm00001d002870 | hypothetical protein |
|  |  |  |  |  | Zm00001d002871 | abcg11 - ABC transporter G family member 11 |
|  |  |  |  |  | Zm00001d002872 | hypothetical protein |
|  |  |  |  |  | Zm00001d002873 | UPF0426 protein chloroplastic |
|  |  |  |  |  | Zm00001d002874 | OSJNBa0038O10.24-like protein |
|  |  |  |  |  | Zm00001d002875 | mneap3 - Maize Nuclear Envelope-Associated Protein3 |
|  |  |  |  |  | Zm00001d002876 | Regulator of chromosome condensation (RCC1) family with FYVE zinc finger domain |
|  |  |  |  |  | Zm00001d002878 | putative ACR |
|  |  |  |  |  | Zm00001d002879 | hypothetical protein |
|  |  |  |  |  | Zm00001d002880 | 3-isopropylmalate dehydrogenase |
|  |  |  |  |  | Zm00001d002881 | Putative pentatricopeptide repeat-containing prote |
|  |  |  |  |  | Zm00001d002882 | SKP1-interacting partner 15 |
|  |  |  |  |  | Zm00001d002885 | uncharacterized |
| Stover Yield | S3_175552327 | 175552327 | 3 | 3.06 | Zm00001d042709 | NPL4-like protein 1 |
|  |  |  |  |  | Zm00001d042710 | hypothetical protein |
|  |  |  |  |  | Zm00001d042711 | Aspartic proteinase A1 |
|  |  |  |  |  | Zm00001d042712 | hypothetical protein |
|  |  |  |  |  | Zm00001d042713 | DNA polymerase alpha catalytic subunit |
|  |  |  |  |  | Zm00001d042714 | Protein S-acyltransferase 8 |
|  |  |  |  |  | Zm00001d042716 | hypothetical protein |
|  |  |  |  |  | Zm00001d042717 | ereb174 - AP2-EREBP-transcription factor 174 |
|  |  |  |  |  | Zm00001d042718 | Protein LIKE COV 2 |
|  |  |  |  |  | Zm00001d042720 | Sterol 3-beta-glucosyltransferase |
|  |  |  |  |  | Zm00001d042721 | bzip95 - bZIP-transcription factor 95 |
|  |  |  |  |  | Zm00001d042722 | hypothetical protein |
|  |  |  |  |  | Zm00001d042723 | ω-hydroxypalmitate O-feruloyl transferase |
|  |  |  |  |  | Zm00001d042724 | TUB-transcription factor 2 |
|  |  |  |  |  | Zm00001d042725 | Sm-like protein LSM3B |
|  |  |  |  |  | Zm00001d042726 | Protein BOLA2 |
|  |  |  |  |  | Zm00001d042727 | fructose-1,6-bisphosphatase |
|  |  |  |  |  | Zm00001d042729 | VAMP-like protein YKT61 |
| Stover Yield | S4_150637846 | 150637846 | 4 | 4.05 | Zm00001d051313 | Leucine-rich repeat (LRR) family protein |
|  |  |  |  |  | Zm00001d026916 | uncharacterized |
|  |  |  |  |  | Zm00001d051318 | Sec14p-like phosphatidylinositol transfer family protein |
|  |  |  |  |  | Zm00001d051320 | esterase/lipase/thioesterase family protein |
|  |  |  |  |  | Zm00001d051321 | ATP-dependent zinc metalloprotease FTSH 7 chloroplastic |
|  |  |  |  |  | Zm00001d051322 | hypothetical protein |
|  |  |  |  |  | Zm00001d051323 | putative receptor-like protein kinase |
|  |  |  |  |  | Zm00001d051324 | chr167 - chromatin complex subunit A 167 |
|  |  |  |  |  | Zm00001d051325 | hypothetical protein |
|  |  |  |  |  | Zm00001d051328 | wrky98 - WRKY-transcription factor 98 |
| Stover Yield | S10_141557921 | 141557921 | 10 | 10.06 | Zm00001d026259 | hypothetical protein |
|  |  |  |  |  | Zm00001d026260 | hypothetical protein |
|  |  |  |  |  | Zm00001d026261 | Core-2/I-branching beta-16-N-acetylglucosaminyltransferase family protein |
|  |  |  |  |  | Zm00001d026262 | SAUR-like auxin-responsive protein family |
|  |  |  |  |  | Zm00001d026263 | Nuclear pore complex protein NUP88 |
|  |  |  |  |  | Zm00001d026265 | 26S proteasome non-ATPase regulatory subunit 1 homolog B |
|  |  |  |  |  | Zm00001d026266 | putative nucleoredoxin 3 |
|  |  |  |  |  | Zm00001d026270 | phd9 - PHD-transcription factor 9 |
| SACC | S6_163628712 | 163628712 | 6 | 6.07 | Zm00001d038960 | S-adenosyl-L-methionine-dependent methyltransferase superfamily protein |
|  | S6_163830244 | 163830244 | 6 | 6.07 | Zm00001d038961 | Kinesin-like protein KIN-10C |
|  |  |  |  |  | Zm00001d038963 | DUF1639 family protein |
|  |  |  |  |  | Zm00001d038964 | hypothetical protein |
|  |  |  |  |  | Zm00001d038965 | purine permease |
|  |  |  |  |  | Zm00001d038966 | Transcription factor bHLH28 |
|  |  |  |  |  | Zm00001d038967 | 6-deoxotyphasterol C-23 hydroxylase |
|  |  |  |  |  | Zm00001d038968 | Eukaryotic aspartyl protease family protein |
|  |  |  |  |  | Zm00001d038969 | Eukaryotic aspartyl protease family protein |
|  |  |  |  |  | Zm00001d038970 | ARM repeat superfamily protein |
|  |  |  |  |  | Zm00001d038971 | MACPF domain-containing protein NSL1 |
|  |  |  |  |  | Zm00001d038972 | putative ubiquitin-conjugating enzyme E2 24 |
|  |  |  |  |  | Zm00001d038973 | 50S ribosomal protein L21 mitochondrial |
|  |  |  |  |  | Zm00001d038974 | alpha/beta-Hydrolases superfamily protein |
|  |  |  |  |  | Zm00001d038975 | Ribosomal protein L1p/L10e family |
|  |  |  |  |  | Zm00001d038976 | cytochrome c oxidase 19-2 |
|  |  |  |  |  | Zm00001d038977 | octanoyl transferase |
|  |  |  |  |  | Zm00001d038978 | AFP homolog 2 |
|  |  |  |  |  | Zm00001d038979 | ARM repeat superfamily protein |
|  |  |  |  |  | Zm00001d038980 | iaa45 - auxin-responsive Aux/IAA family member 45 |

a: SACC: Saccharification efficiency

b: The number before the underscores indicates the chromosome number and the number after the underscore indicates the physical position in bp within the chromosome.

c: Chromosome

d: A bin is the interval that includes all loci from the leftmost or top Core Marker to the next Core Marker. The genetic maps are divided into 100 segments of approximately 20 centiMorgans designated with the chromosome number followed by a two-digit decimal ^24^
