# Supplementary material for: Association mapping for maize stover yield and saccharification efficiency using a multiparent advanced generation intercross (MAGIC) population
Source: Sci Rep. 2021 Feb 9;11:3425. doi: 10.1038/s41598-021-83107-1 (PMC7873224; doi:10.1038/s41598-021-83107-1)
Supplement: Supplementary file 7 — Supplementary Information 7. [file 41598_2021_83107_MOESM7_ESM.pdf]

Association Mapping for Maize Stover Yield and Saccharification Efficiency Using a Multi-Parent Advanced Generation Intercross (MAGIC) Population

López-Malvar, A[1]*; Butron A[3]; Malvar, RA[3]; Gómez, LD[2]; Faas, L[2]; McQueen-Mason, S[2]; Revilla P[3]; Figueroa-Garrido, DJ[1]; Santiago, R[1]

[1]Facultad de Biología, Departamento de Biología Vegetal y Ciencias del Suelo, Universidad de Vigo, As Lagoas Marcosende, Vigo 36310, Spain. Agrobiología Ambiental, Calidad de Suelos y Plantas (UVIGO), Unidad Asociada a la MBG (CSIC);

[2]Misión Biológica de Galicia (CSIC), Pazo de Salcedo, Carballeira 8, 36143, Spain

[3]CNAP, Department of Biology, University of York, Heslington, York YO10 5YW, UK

*Corresponding author: alopezmalvar@uvigo.es

Supplementary Figure 1: Decay of LD (R^2) as a function of distance (bp) in a 10 Mbp window. Regression line is based on Hill and Weir (1988)

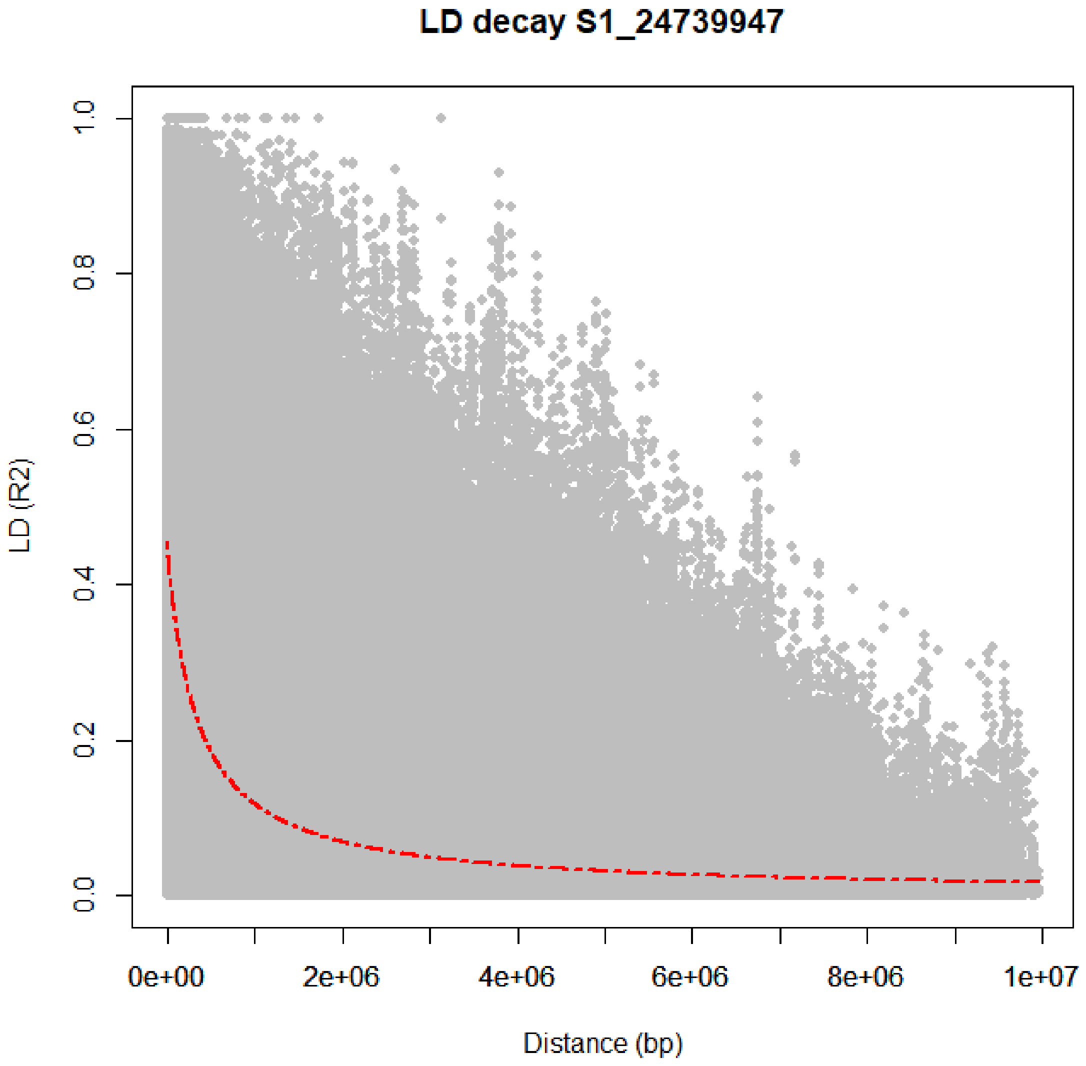

LD decay S1_24739947
LD (R2)
1.0
0.8
0.6
0.4
0.2
0.0
0e+00
2e+06
4e+06
6e+06
8e+06
1e+07
Distance (bp)

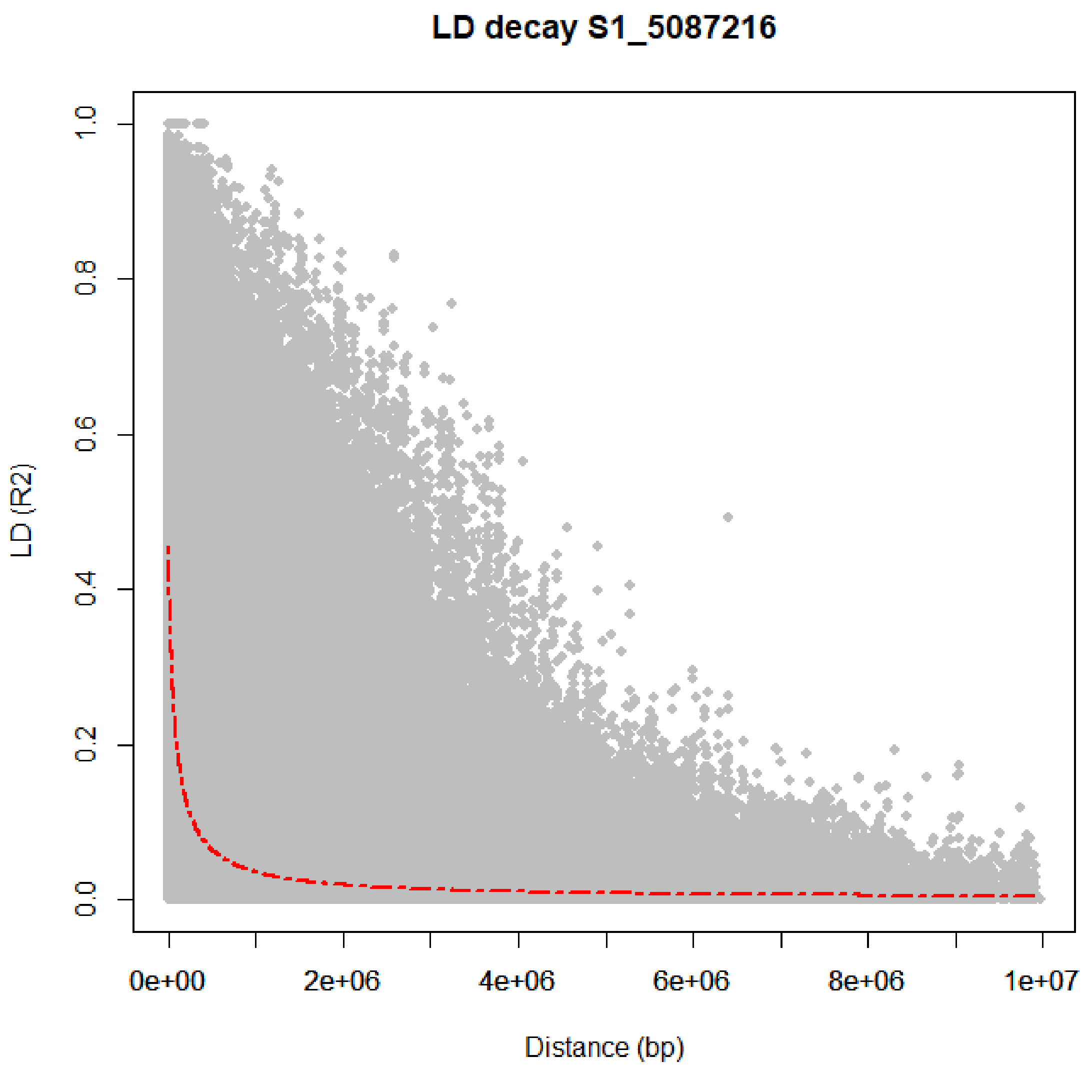

LD decay S1_5087216
LD (R2)
0.0
0.2
0.4
0.6
0.8
1.0
0e+00
2e+06
4e+06
6e+06
8e+06
1e+07
Distance (bp)

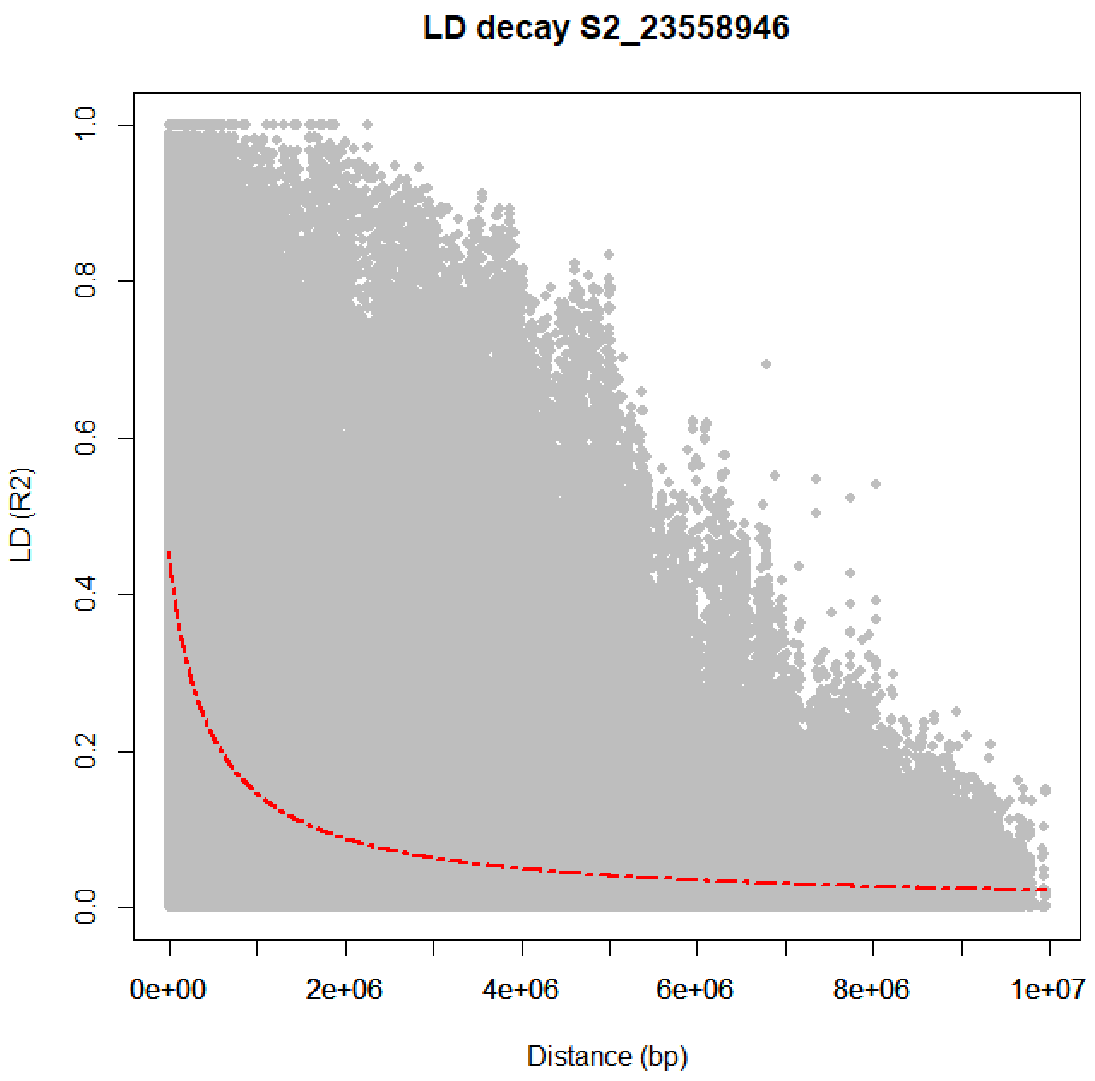
LD decay S2_23558946
LD (R2)
1.0
0.8
0.6
0.4
0.2
0.0
0e+00
2e+06
4e+06
6e+06
8e+06
1e+07
Distance (bp)

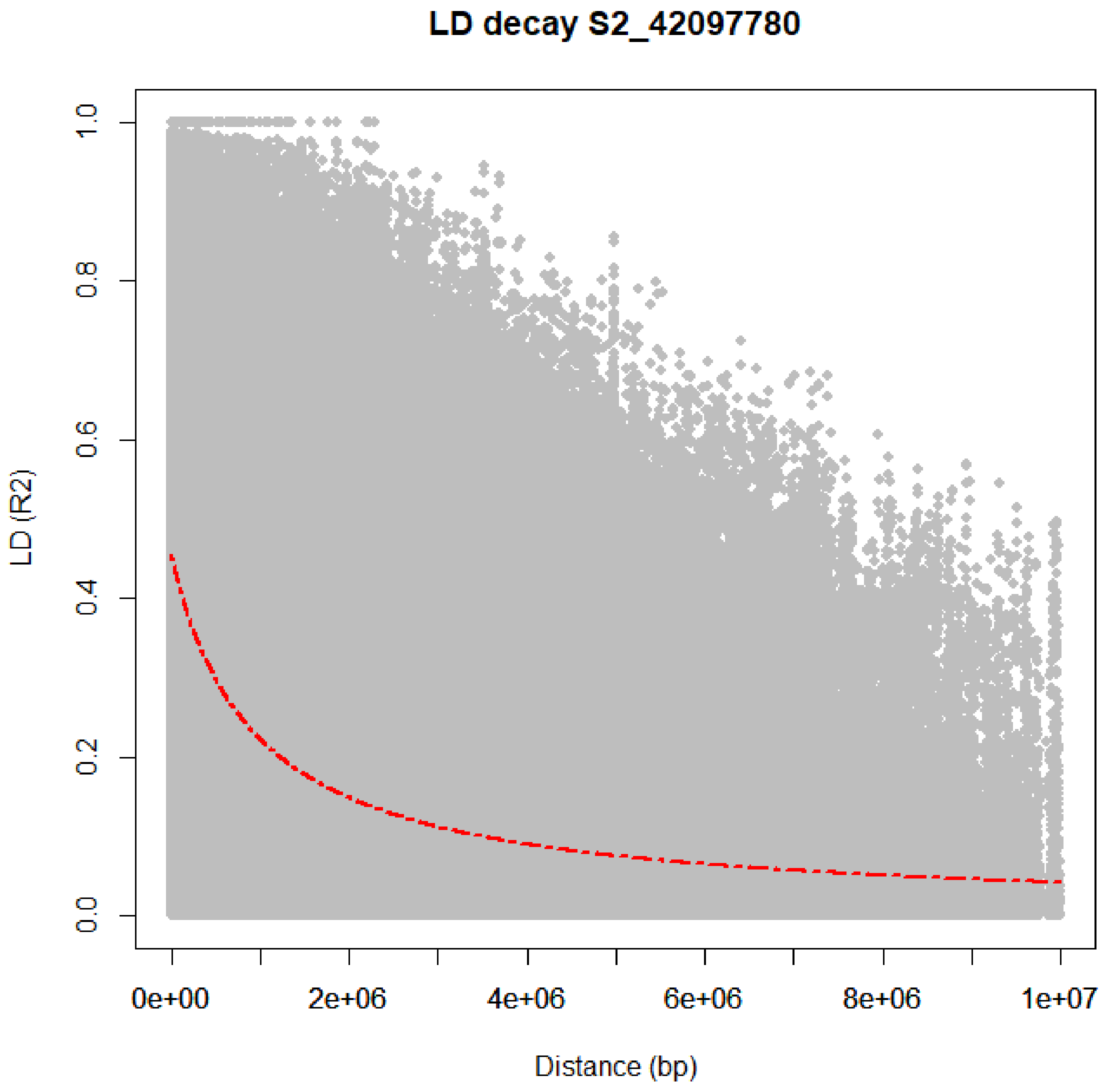
LD decay S2_42097780
LD (R2)
0.0
0.2
0.4
0.6
0.8
1.0
0e+00
2e+06
4e+06
6e+06
8e+06
1e+07
Distance (bp)

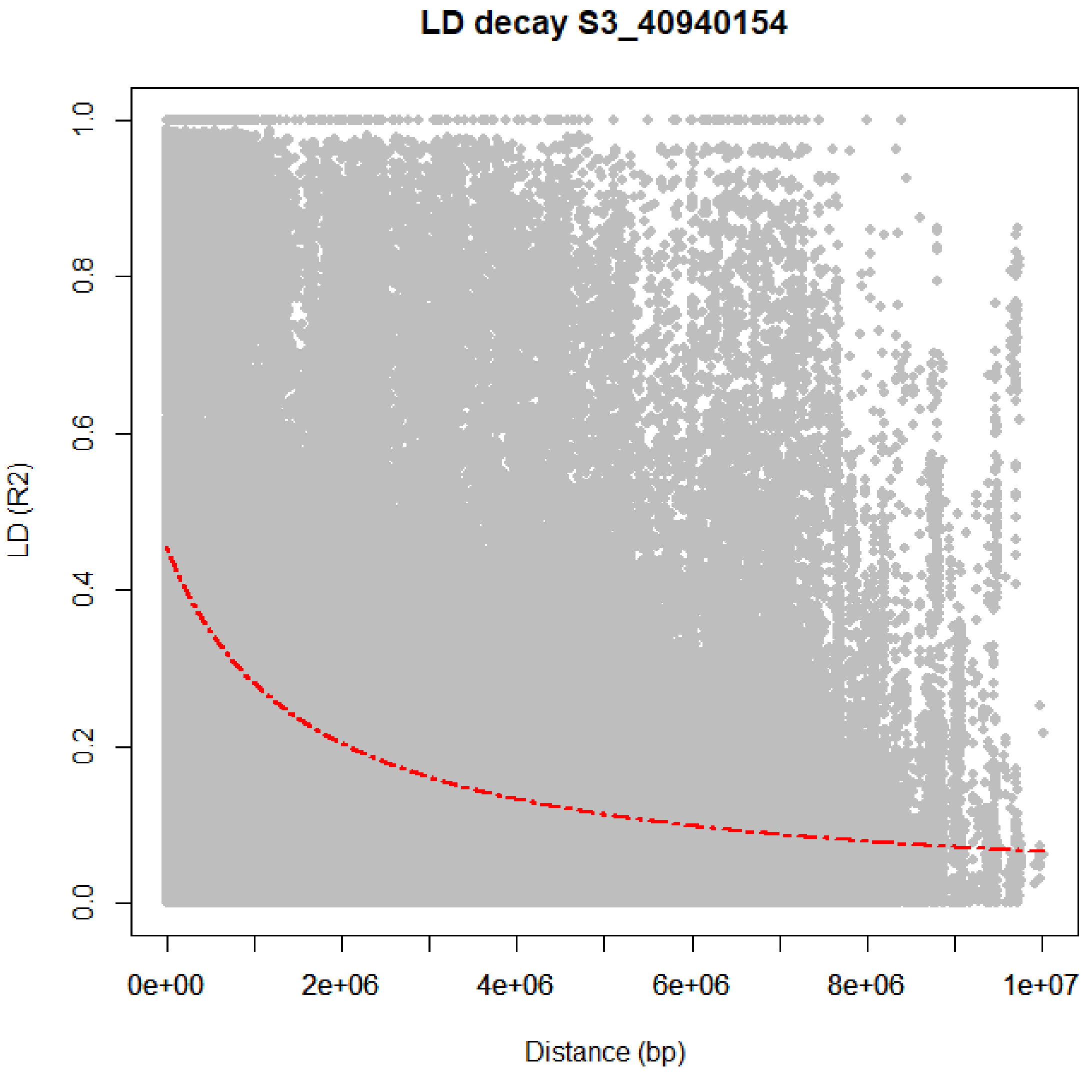
LD decay S3_40940154
LD (R2)
0.0
0.2
0.4
0.6
0.8
1.0
0e+00
2e+06
4e+06
6e+06
8e+06
1e+07
Distance (bp)

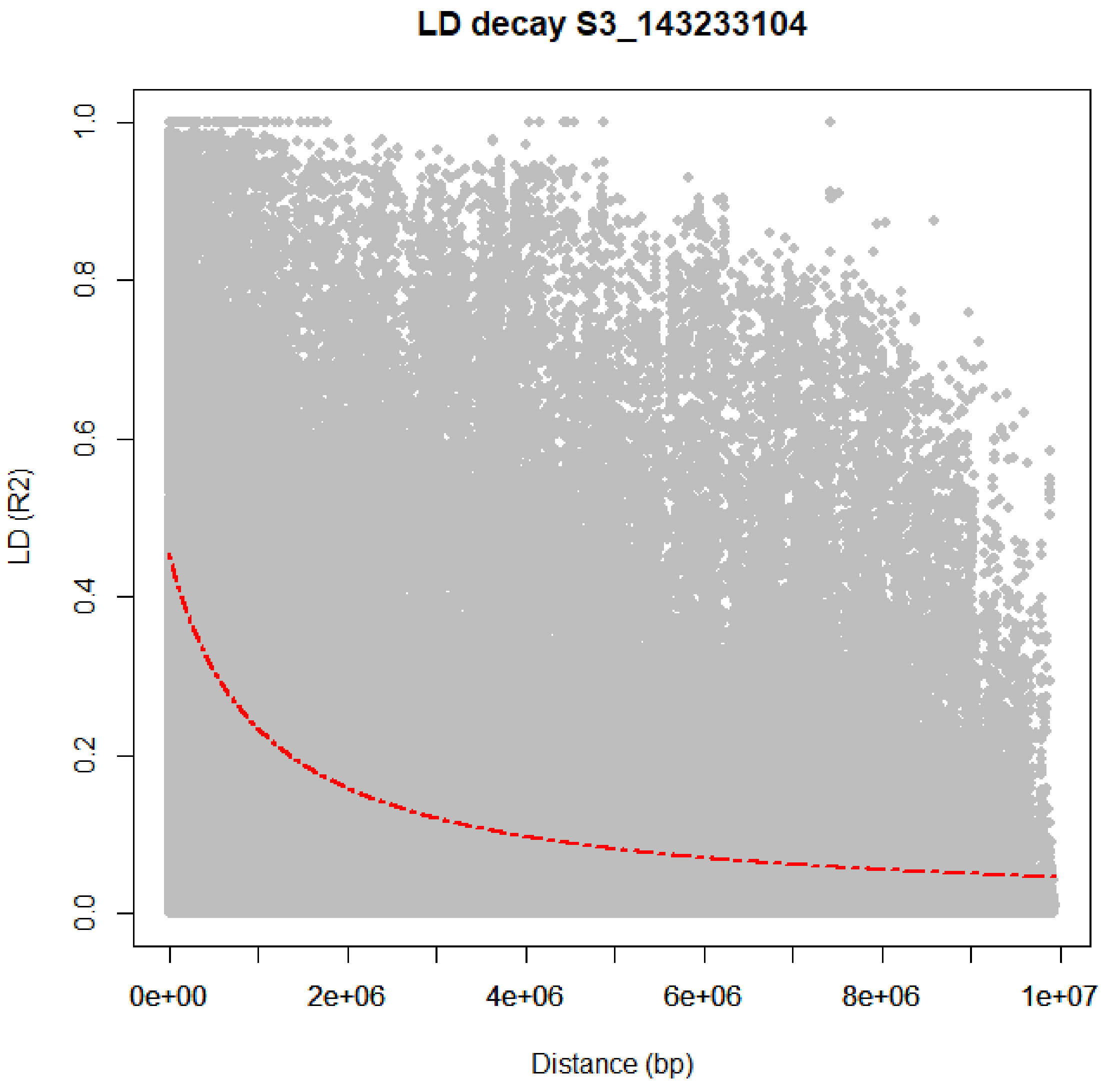
LD decay S3_143233104
LD (R2)
1.0
0.8
0.6
0.4
0.2
0.0
0e+00
2e+06
4e+06
6e+06
8e+06
1e+07
Distance (bp)

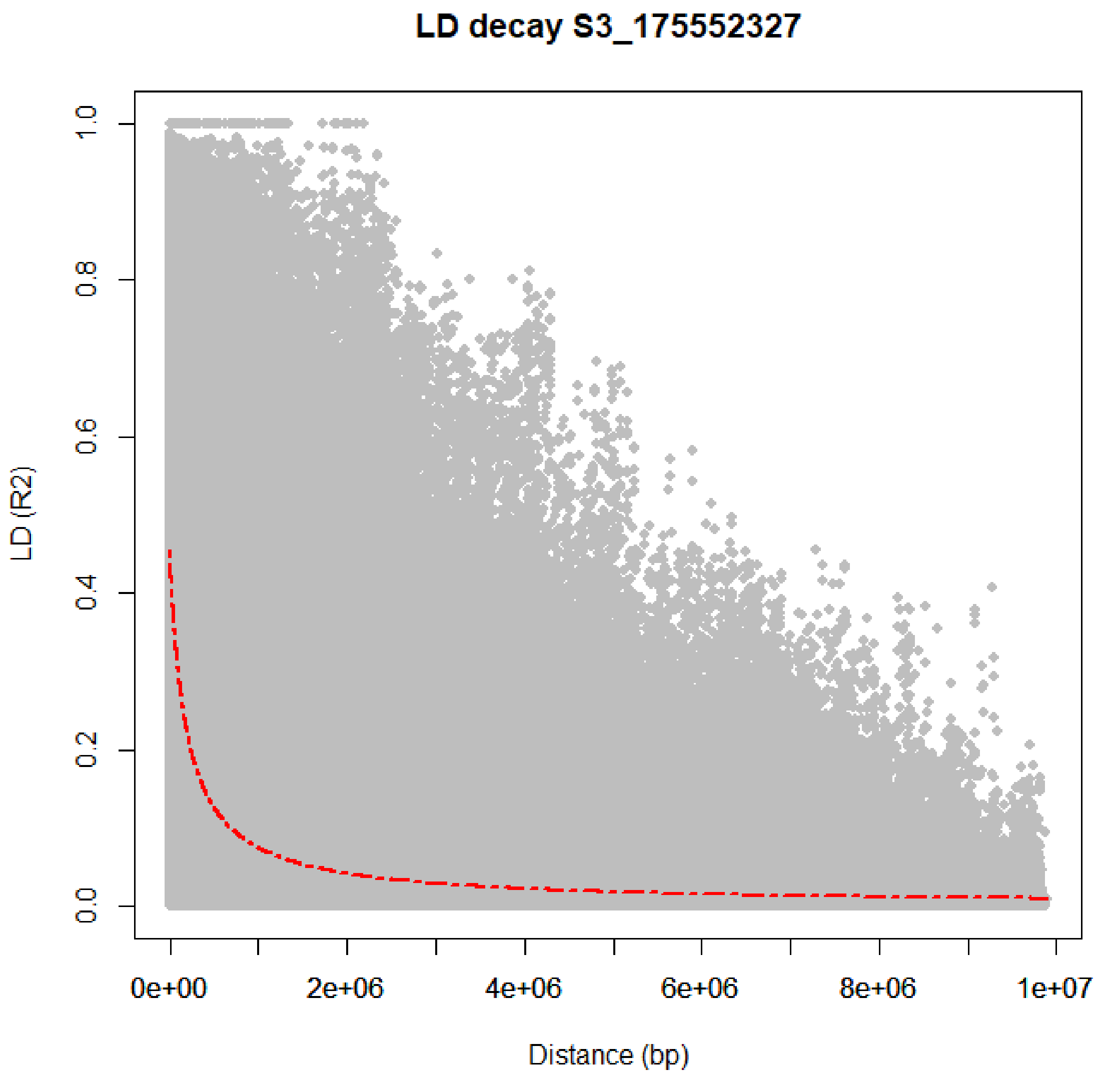
LD decay S3_175552327
LD (R2)
0.0
0.2
0.4
0.6
0.8
1.0
0e+00
2e+06
4e+06
6e+06
8e+06
1e+07
Distance (bp)

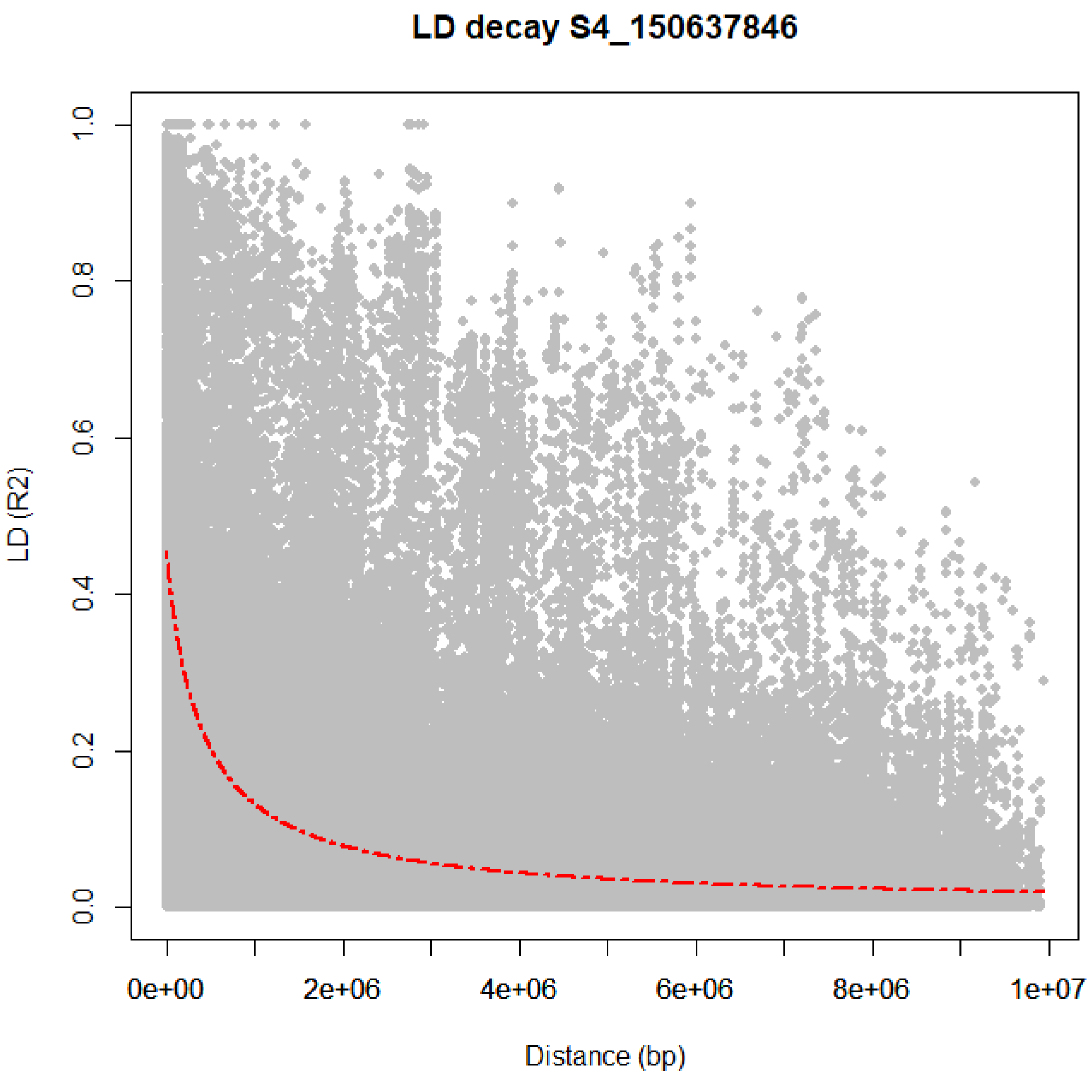
LD decay S4_150637846
LD (R2)
1.0
0.8
0.6
0.4
0.2
0.0
0e+00
2e+06
4e+06
6e+06
8e+06
1e+07
Distance (bp)

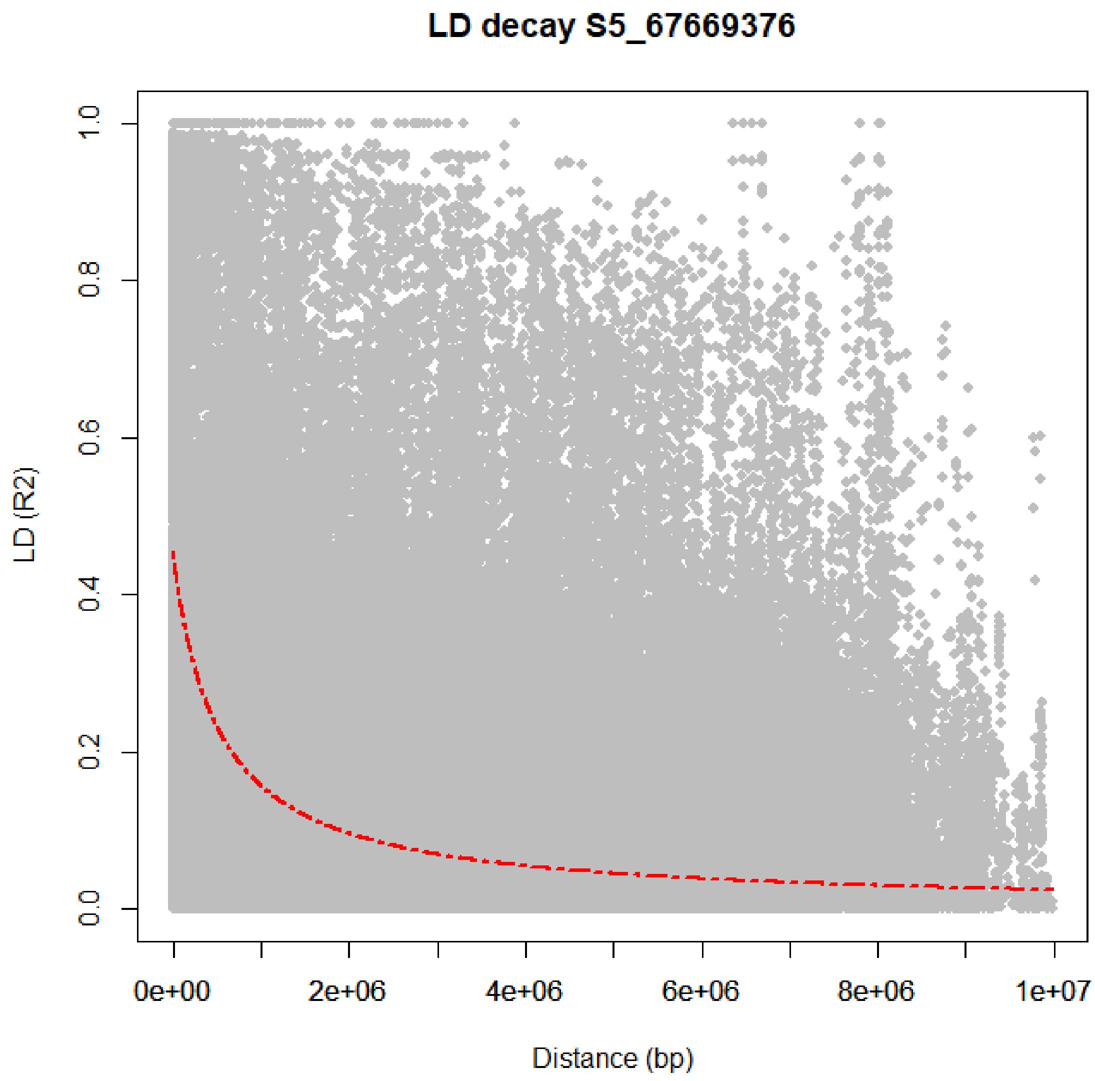
LD decay S5_67669376
LD (R2)
1.0
0.8
0.6
0.4
0.2
0.0
0e+00
2e+06
4e+06
6e+06
8e+06
1e+07
Distance (bp)

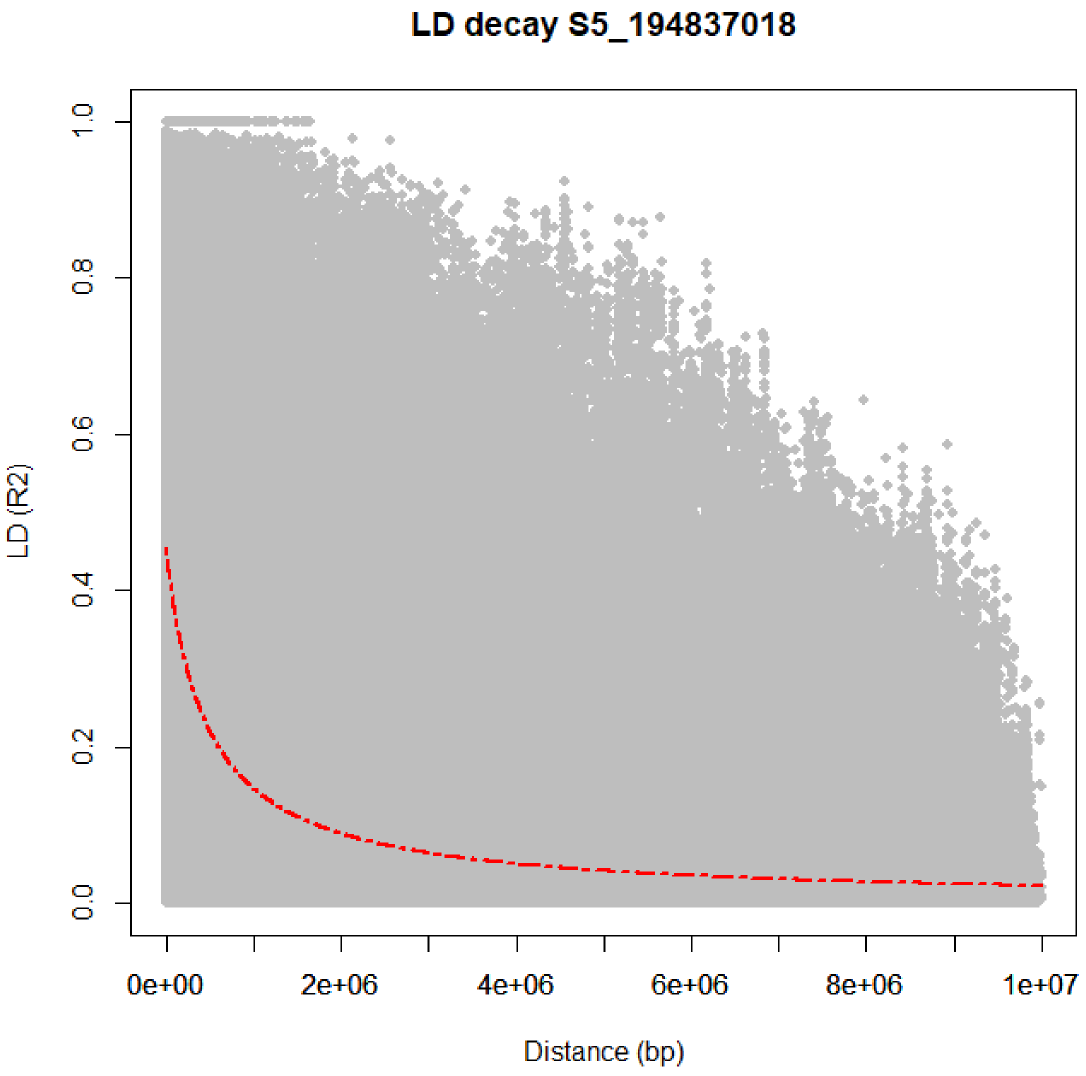

LD decay S5_194837018
LD (R2)
0.0
0.2
0.4
0.6
0.8
1.0
0e+00
2e+06
4e+06
6e+06
8e+06
1e+07
Distance (bp)

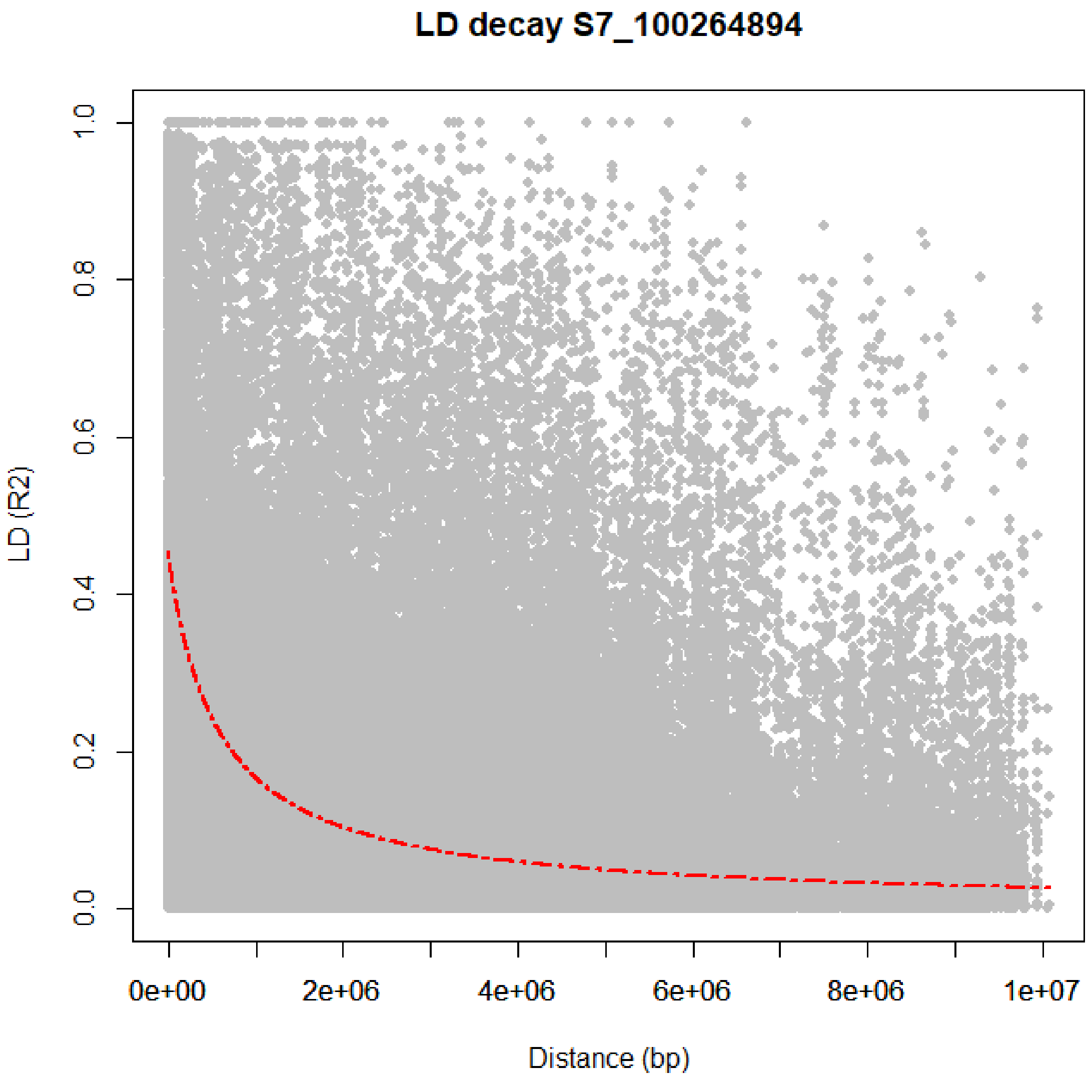
LD decay S7_100264894
LD (R2)
1.0
0.8
0.6
0.4
0.2
0.0
0e+00
2e+06
4e+06
6e+06
8e+06
1e+07
Distance (bp)

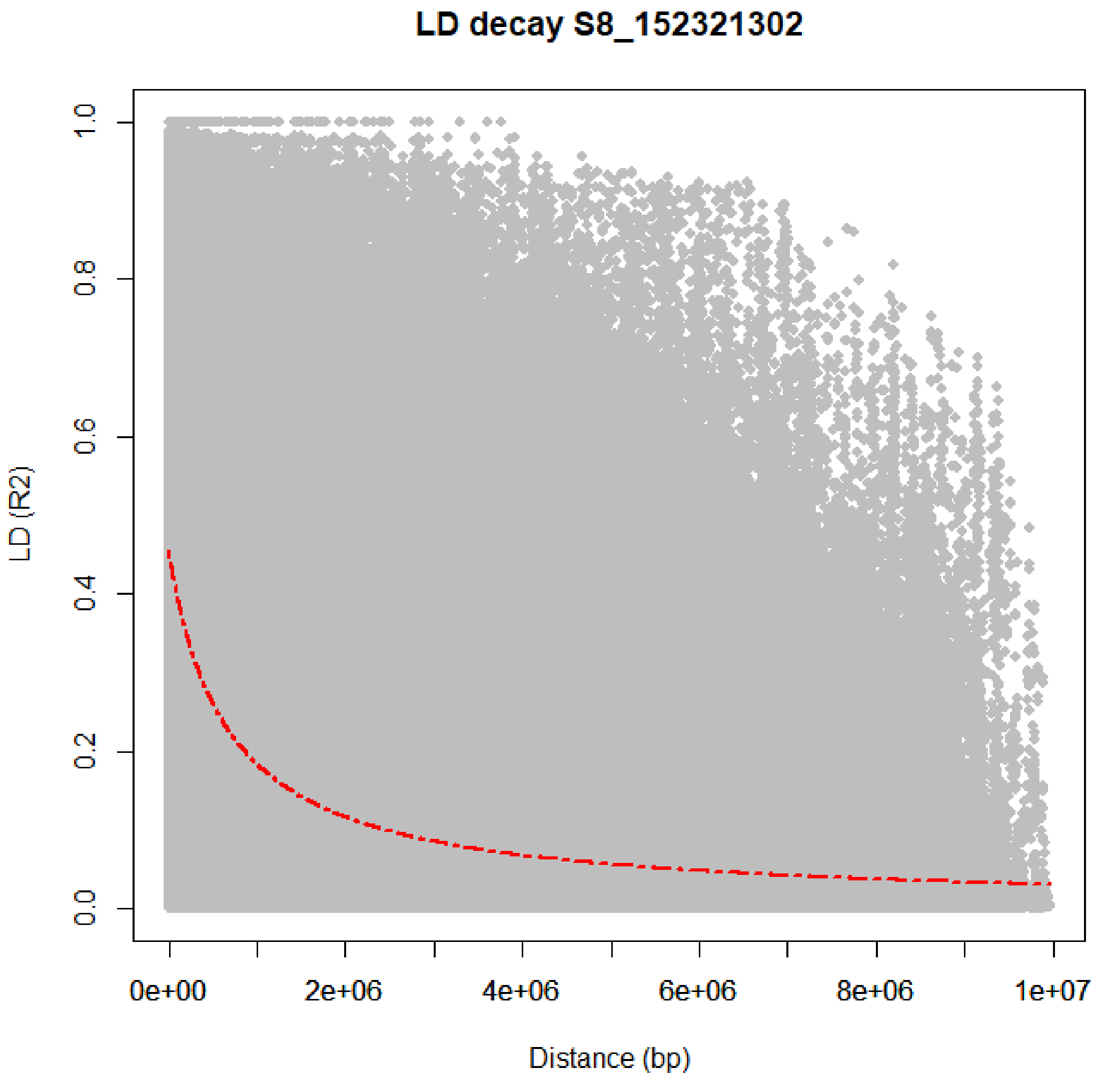

LD decay S8_152321302
LD (R2)
1.0
0.8
0.6
0.4
0.2
0.0
0e+00
2e+06
4e+06
6e+06
8e+06
1e+07
Distance (bp)

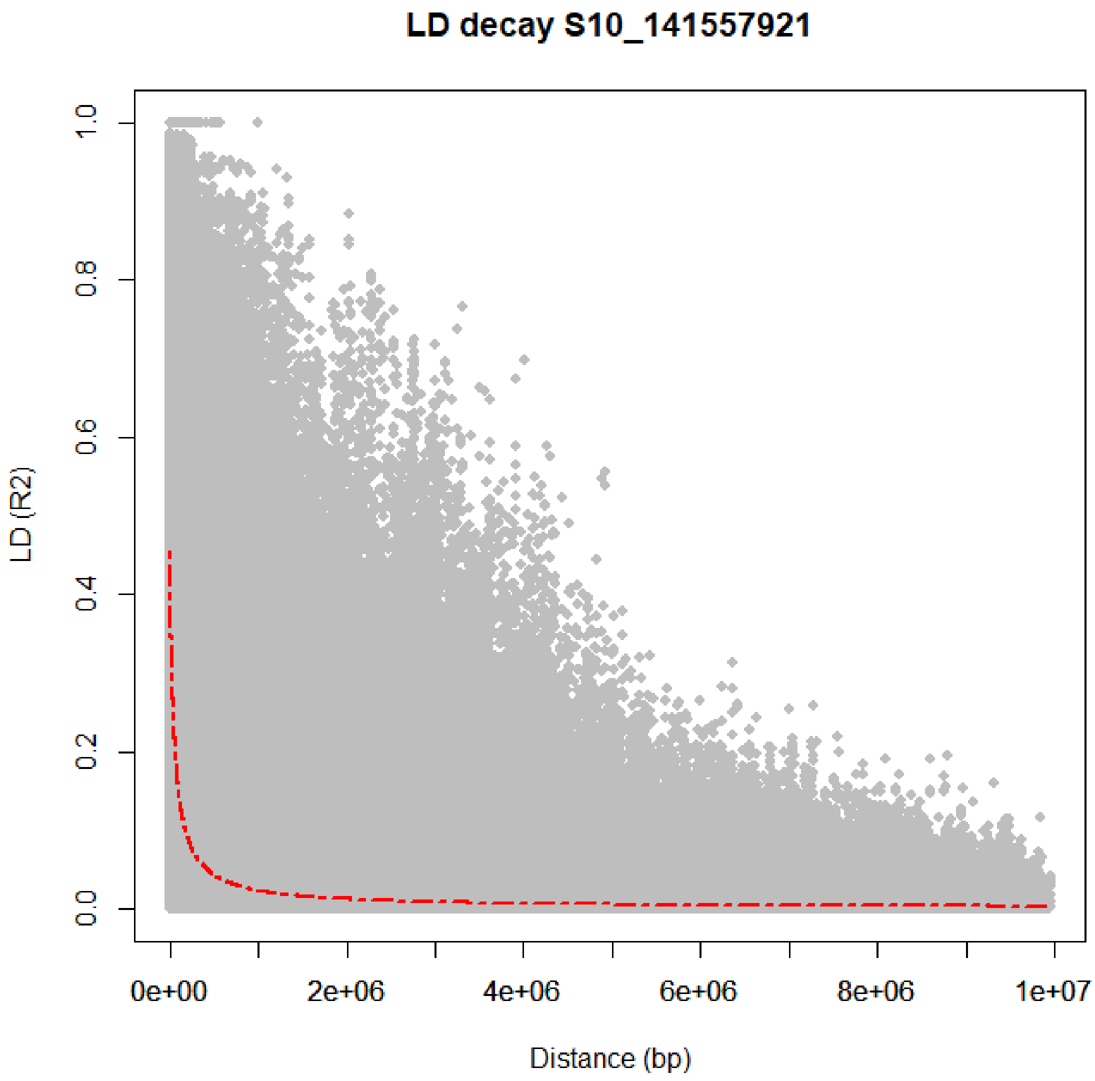
LD decay S10_141557921
LD (R2)
1.0
0.8
0.6
0.4
0.2
0.0
0e+00
2e+06
4e+06
6e+06
8e+06
1e+07
Distance (bp)

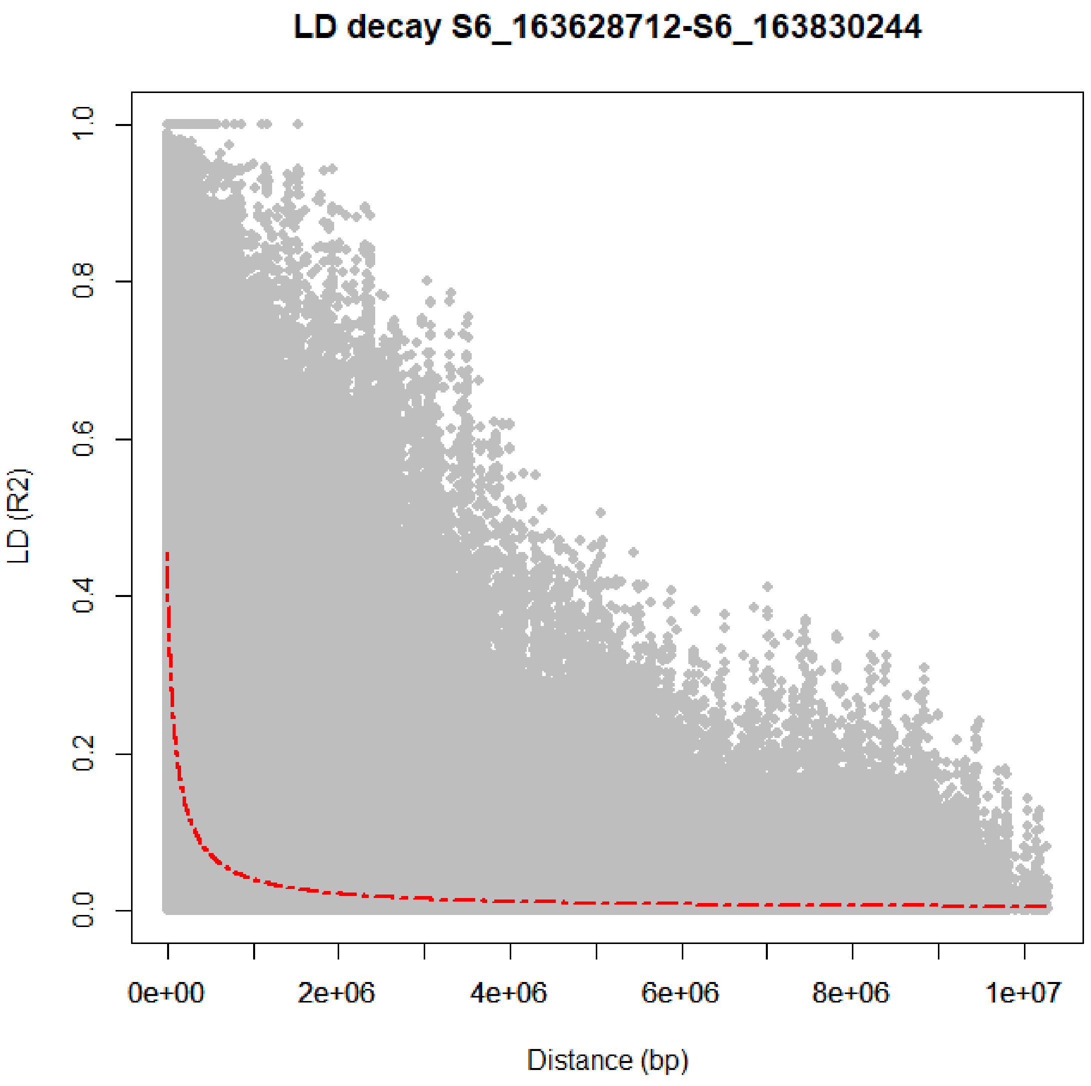
LD decay S6_163628712-S6_163830244
LD (R2)
0.0
0.2
0.4
0.6
0.8
1.0
0e+00
2e+06
4e+06
6e+06
8e+06
1e+07
Distance (bp)
